# Current Evidence, Challenges, and Opportunities of Physiologically Based Pharmacokinetic Models of Atorvastatin for Decision Making

**DOI:** 10.3390/pharmaceutics13050709

**Published:** 2021-05-13

**Authors:** Javier Reig-López, Alfredo García-Arieta, Víctor Mangas-Sanjuán, Matilde Merino-Sanjuán

**Affiliations:** 1 Department of Pharmacy and Pharmaceutical Technology and Parasitology, University of Valencia, 46010 Valencia, Spain; jareiglo@alumni.uv.es (J.R.-L.); Matilde.Merino@uv.es (M.M.-S.); 2 División de Farmacología y Evaluación Clínica, Departamento de Medicamentos de Uso Humano, Agencia Española de Medicamentos y Productos Sanitarios, Calle Campezo 1, Edificio 8, 28022 Madrid, Spain; agarciaa@aemps.es; 3 Interuniversity Research Institute for Molecular Recognition and Technological Development, 46100 Valencia, Spain

**Keywords:** atorvastatin, lactonization, active metabolites, open acid form, P-glycoprotein, solubility

## Abstract

Atorvastatin (ATS) is the gold-standard treatment worldwide for the management of hypercholesterolemia and prevention of cardiovascular diseases associated with dyslipidemia. Physiologically based pharmacokinetic (PBPK) models have been positioned as a valuable tool for the characterization of complex pharmacokinetic (PK) processes and its extrapolation in special sub-groups of the population, leading to regulatory recognition. Several PBPK models of ATS have been published in the recent years, addressing different aspects of the PK properties of ATS. Therefore, the aims of this review are (i) to summarize the physicochemical and pharmacokinetic characteristics involved in the time-course of ATS, and (ii) to evaluate the major highlights and limitations of the PBPK models of ATS published so far. The PBPK models incorporate common elements related to the physicochemical aspects of ATS. However, there are important differences in relation to the analyte evaluated, the type and effect of transporters and metabolic enzymes, and the permeability value used. Additionally, this review identifies major processes (lactonization, P-gp contribution, ATS-Ca solubility, simultaneous management of multiple analytes, and experimental evidence in the target population), which would enhance the PBPK model prediction to serve as a valid tool for ATS dose optimization.

## 1. Introduction

Statins are the first-line treatment of choice/gold-standard in the pharmacological management of hypercholesterolemia [1], and they have been positioned as the most effective oral drugs for the treatment and prevention of cardiovascular diseases associated with dyslipidemia [2,3]. Statins are reversible inhibitors of 3-hydroxy-3-methyl-glutharyl-coenzyme A (HMG-CoA) reductase, the enzyme responsible for de novo cholesterol biosynthesis. Statins can be administered in the active form (atorvastatin, fluvastatin, pitavastatin, pravastatin, and rosuvastatin) or as inactive drugs (simvastatin and lovastatin), which require activation within the organism. Statins are, in general, safe and well tolerated [4,5]. In terms of safety concerns, the most frequent adverse events are myopathies (5–10%) [6], ranging from muscle pain to very rare cases of fatal rhabdomyolysis [7], and hepatotoxicity, which is present in 1 % of treated patients and resolves spontaneously after withdrawal of the drug [5].

Among the statins, atorvastatin (ATS) is one of the most prescribed [8] statin worldwide for the treatment of hypercholesterolemia in order to diminish the cardiovascular risk [9]. ATS is a second-generation synthetic statin that is administered as the calcium salt of the active hydroxy-acid form, although some generics have been developed with the magnesium salt to avoid the patent protection of the calcium salt. According to the desired reduction in low-density lipoprotein cholesterol (LDLc) levels, the clinical posology involves the use of 10–80 mg once daily at any time of the day. Despite its wide, cost-effective use and pharmacological response, several factors undermine the clinical response of statins in the treatment of hypercholesterolemia, involving low adherence of patients, inadequate selection of the active ingredient, polymorphisms, adverse events (myopathies), drug–drug interactions (DDIs), etc. The use of model-based strategies able to properly characterize the time-course of the active entities is encouraged in order to optimize the dosing strategy in patients. Physiologically based pharmacokinetic (PBPK) modelling has emerged as a solid tool in the decision-making process during drug development, which has gained regulatory recognition in the last years [10,11]. The main applications of PBPK models range from drug–drug interactions (DDI), transporter evaluation, food–drug interactions, intrinsic factors evaluation, and extrapolation of drug exposure in special subgroups of patients. Therefore, the aims of this review are (i) to summarize the physicochemical and pharmacokinetic (PK) characteristics involved in the time-course of ATS, and (ii) to evaluate the major highlights and limitations of the PBPK models of ATS published so far.

## 2. Physicochemical Properties

### 2.1. Solubility

ATS (546 g/mol, pK_a_ 4.46) belongs to class II of the Biopharmaceutical Classification System (BCS) due to its low solubility in gastrointestinal fluid, which contributes to its low bioavailability (12%) [12,13,14]. ATS solubility in deionized water is reported to be 0.0206 mg/mL at 37 °C [14]. A tri-hydrated calcium salt form of ATS (ATS-Ca) is included in the commercially available tablets of ATS. ATS-Ca has been isolated in amorphous and crystalline forms, but it is commercialized in its crystalline form due to the higher stability. ATS-Ca solubility increases with pH, being insoluble in acidic aqueous solutions of pH < 4 [15]. Solubility values in aqueous media for the amorphous and crystalline forms at 37 °C are 0.11–0.12 mg/mL in water, 0.01 mg/mL in HCl 0.1 N, and 0.72 and 0.70 mg/mL in sodium phosphate 0.05 M pH 7.4, respectively [16]. Great efforts have been performed to improve ATS oral bioavailability through formulation strategies to increase the solubility and/or dissolution rate of ATS-Ca such as micronization by antisolvent precipitation [15], microcapsulation [17], co-grinding techniques [18], co-amorphous formulations with nicotinamide [19], dry emulsions [13], inclusion complexes [20], and use of drug resinates [21]. Since ATS is administered mainly as the calcium salt, low solubility in the gastrointestinal (GI) tract should be considered in order to assess its PK characteristics.

### 2.2. Lipophilicity

The chemical structure of ATS (and of statins in general) can be divided into three parts: (1) the analogue of the target enzyme substrate (3-hydroxy-3-methyl-glutaril coenzyme A or HMG-CoA), (2) a complex hydrophobic ring structure covalently linked to the substrate analogue, and (3) side groups on the rings that define the solubility and PK properties [1]. While the analogue of the HMG-CoA (the mevalonate-like pharmacophore) is responsible for the reversible inhibition of the HMG-CoA reductase, the ring structure and its substituents lead to differences in lipophilicity, absorption properties, plasma protein binding, and elimination [22]. Lipophilicity of ATS is determined by its logP of 4.1 [22] and its logD at pH 7.4 (1.52) [3].

## 3. Absorption

A rapid oral absorption is expected after ATS administration, since the median T_max_ is reported to be 1 h, with a range of 0.5–3 h [3]. The oral fraction absorbed of ATS is 30% between 10 and 80 mg, and its oral bioavailability is known to be low (12%) [22,23,24,25] and dose-independent. Therefore, dissolution and pre-systemic metabolism (gut wall and liver first-pass effect) are the key relevant processes affecting ATS bioavailability. The rate and extent of ATS absorption are influenced by the time of administration [26] and the presence of food [23]. A study with 16 healthy volunteers revealed that ATS maximum plasma concentration (C_max_) and area under the plasma concentration-time curve (AUC) diminished by 47.9% and 12.7%, respectively, when an 80 mg dose was administered with food [27]. This reduction in ATS exposure has also been reported at the lowest dose level (10 mg) [28]. In this sense, the administration of ATS with food decreases its bioavailability by 13% [22,23]. Despite the administration of ATS in the evening is associated with a lower exposure compared to when it is dosed in the morning (with mean C_max_ and AUC values 31% and 57% lower, respectively), and a food effect has been determined, no difference in the clinical response is observed [26,28]. For this reason, ATS can be administered at any time of the day and without regard to food. Gender is another covariate influencing ATS exposure, but it lacks any clinical relevance, despite the 10% lower AUC and 20% higher C_max_ in females compared to males [12].

The intestinal absorption of ATS is a complex process, as the net transport of this drug through the gut wall involves multiple mechanisms, being not only restricted to passive diffusion. In vitro experiments in Caco-2 cell monolayers revealed an apparent permeability (P_app_) in the basolateral-to-apical direction 7-fold higher than in the apical-to-basolateral direction, showing the role of P-glycoprotein (P-gp) efflux (K_m_ and J_max_ values of 115 ± 19 µM and 141 ± 11 pmol/cm^2^/min, respectively [29]). The interaction of ATS and P-gp has also been demonstrated in Madin–Darby canine kidney cells (MDCK) expressing human P-gp [30]. In this cell line, the efflux ratio after correcting with parental MDCKII cells resulted in 4.46 for ATS acid, suggesting ATS acid as a moderate substrate of P-gp. Moreover, monocarboxylic acid co-transporter (MCT) has been identified as a relevant transporter in the ATS absorption from the gut lumen with a K_m_ value in the mM range. As clinically relevant maximal concentrations in the intestinal lumen are estimated to be within the 70–550 µM range after doses of 10 to 80 mg [3], ATS MCT-mediated absorption may be a linear process at this concentration, which is consistent with the proportional increase in the extent (AUC) of ATS absorption in the 10 to 80 mg dose range.

## 4. Distribution

The passive membrane permeability of statins increases along with their lipophilicity, and, consequently, lipophilic statins are distributed into peripheral tissues [31]. ATS has a volume of distribution of 5.4 L/kg [24] and exhibits a high degree of plasma protein binding (>98%) [32]. Statin accumulation in the liver is mediated by hepatic uptake through the organic anion transporting polypeptide (OATP) family, sodium-dependent taurocholate co-transporting polypeptide (NTCP), and by efflux transporters of the ATP-binding cassette (ABC) family, located on the basolateral and canalicular membranes of the liver, respectively [33]. In vitro kinetic studies on ATS hepatic uptake revealed that OATP1B1 and OATP1B3 were the major ATS uptake transporters, while NTCP was found to be of minor importance in ATS disposition. The average contribution to ATS uptake resulted as OATP1B1 > OATP1B3 >> OATP2B1 > NTCP; their respective K_m_ (µM) values are 0.77, 0.73, 2.84, and 185 and V_max_ (pmol/min/mg protein) values are 6.61, 2.29, 24.27, and 2260, respectively [34]. An ATS intrinsic uptake clearance of 2030 mL/min (95% CI: 1140–2620 mL/min) was predicted and, assuming the same passive diffusion across the cell membrane of hepatocytes and HEK293 cells (120 µL/min/g of liver), transporter-mediated active uptake of ATS dominates overall ATS hepatic uptake [34]. Moreover, polymorphisms in transporter genes have been reported to affect the PK of statins and their therapeutic effects [35,36]. It has been demonstrated that the liver-to-plasma concentration ratio of ATS is 2.7-fold higher (*p* = 0.002) in wild-type when compared to *Slco1b2−*/*−* mice after 1 mg/kg ATS tail vein injection [33]. In humans, it has been observed that ATS and its metabolites are sensitive to polymorphisms in *SLCO1B1*, as plasma concentrations were higher in subjects carrying the reduced function *SLCO1B1* 521C allele (T/C genotype) compared with the wild-type subjects (521 T/T) [37]. Another example comes from a fixed-order crossover study in 660 Finnish healthy volunteers [35], which concluded that individuals carrying the *ABCG2* c.421C > A single-nucleotide polymorphism (SNP) had a 72% higher ATS AUC_0-inf_ than individuals with the c.421CC genotype (*p* = 0.049), suggesting that the *ABCG2* polymorphisms affect the PK of ATS. As the elimination half-life was not influenced by *ABCG2* polymorphism, it allowed the authors to conclude that ABCG2 influences mostly during the absorption phase, enhancing ATS absorption and bioavailability [35].

## 5. Metabolism

**Figure 1:**
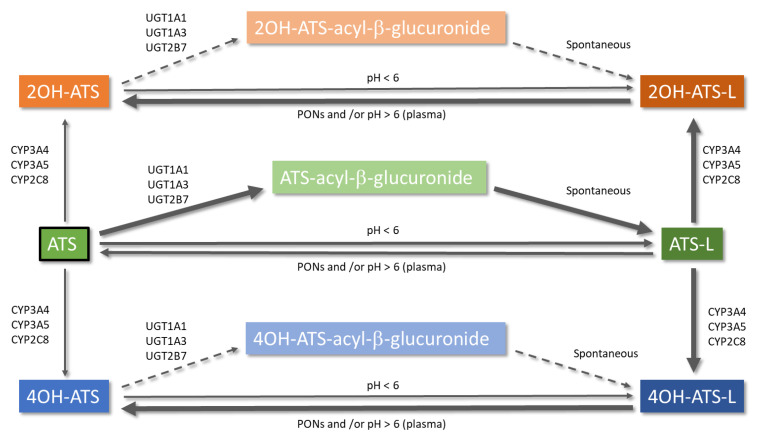
Metabolic pathways of atorvastatin. Arrow thickness informs directly about the relevance of the reaction and the sense of the equilibrium. Dashed arrows represent a theoretically possible lactonization of 2OH-ATS and 4OH-ATS via an acyl-β-glucuronide. ATS: atorvastatin open acid form; ATS-L: atorvastatin lactone form.

Metabolism of ATS is an intricate pathway of different reactions that include glucuronidation [7,8,38], lactonization [39], and cytochrome P450-mediated oxidation [40,41]. A simplified scheme with the different metabolic pathways of ATS is depicted in Figure 1. ATS is administered as the hydroxy acid form (calcium salt), and its active metabolites (*ortho*-hydroxy atorvastatin (2OH-ATS) and *para*-hydroxy atorvastatin (4OH-ATS)) are equipotent to the parent compound, being responsible for 70% of the HMG-CoA reductase inhibitory activity of ATS [42]. The in vitro HMG-CoA reductase inhibitory activity (IC_50_) values for ATS, 2OH-ATS, and 4OH-ATS are 3.71, 5.54, and 3.29 nM, respectively [43]. Both metabolites, as the parent compound, are equilibrated with the corresponding lactone forms (ATS-L, 2OH-ATS-L, and 4OH-ATS-L) [38,39,41]. It has been demonstrated that lactonization might occur non-enzymatically at pH < 6 [44] or enzymatically, being the former pathway negligible at pH > 6. The formation of an acyl-glucuronide prior to lactonization is expected to be the major pathway for the enzymatic lactonization of ATS in humans, which is catalyzed by UDP-glucuronosyltransferases (UGTs) UGT1A1, UGT1A3, and UGT2B7. The isoenzyme UGT1A3 is the major contributor to this process with 200 times more activity than UGT2B7 [7]. The mechanism proposed for the lactonization is the formation of an acyl-β-D-glucuronide conjugate of the ATS acid (parent), elimination of the glucuronic moiety, and final spontaneous cyclization to the corresponding lactone [38]. ATS glucuronidation, and thus lactonization, follows non-linear kinetics with K_m_ values of 4 and 20 µM and V_max_ values of 2280 and 120 pmol/min/mg for UGT1A3 and UGT2B7, respectively [7]. ATS lactonization is affected by polymorphisms in the *UGT1A* locus and has been demonstrated both in vitro and in vivo in healthy volunteers [8]. On the other hand, the hydrolysis of the lactone forms of ATS and its metabolites to the corresponding carboxylates takes place non-enzymatically at pH > 6 [44] or can be catalyzed by plasmatic esterases or paraoxonases (PONs) [38]. PONs are a family of esterase/lactonase enzymes whose encoding genes are located in tandem in the long arm of human chromosome 7 (7q 21–22) [45], and PON1, PON2, and PON3 are highly involved in ATS metabolism. In addition, ATS increases the expression of PON2 [46]. A 3.8-fold higher ATS-L hydrolysis rate through PON1 and PON3 has been demonstrated in vitro when compared to spontaneous hydrolysis in a pooled microsomal fraction [47]. Additionally, results from incubation experiments in human liver microsomes (HLMs) show a median ATS formation rate through hydrolysis of the corresponding lactone of 309.70 pmol/min/mg protein [47]. Hydrolysis of lactone forms has been demonstrated to occur in plasma [48]. Therefore, this process must be considered when modelling ATS and its metabolites to better assess its pharmacokinetics.

Cytochrome P450-mediated oxidative metabolism has been described as a major pathway of biotransformation for statins in humans [38], where CYP3A4 is the major enzyme involved in the formation of the two hydroxy-metabolites of ATS [39,41]. The CYP3A4-mediated oxidation is clearly polarized to the lactone forms, with K_m_ values of 3.9 and 1.4 µM and V_max_ values of 4235 and 14312 pmol/min/mg for the ortho- and para-hydroxylated metabolites, respectively [39]. Differences in K_m_ and V_max_ values between the acid and lactone form of ATS result in an intrinsic clearance ratio lactone/acid equal to 73 [40] and in specific metabolite clearance ratios for ortho-hydroxylation and para-hydroxylation of 20.2 and 83.1, respectively [39]. Quantum mechanics/molecular mechanics (QM/MM) have revealed that the acid form of ATS must pay a desolvation penalty of 5 Kcal/mol to enter in the more hydrophobic active site of the enzyme [39]. Moreover, the higher V_max_ value for the para-hydroxylation of ATS-L has been attributed to a shorter distance to the heme oxygen atom of CYP3A4 [39]. Inhibition studies have demonstrated that ATS-L could be an inhibitor of the metabolism of the acid form [39]. It could be concluded that ATS lactonization changes its affinity to CYP3A, affecting the preferred hydroxylation positions, and may be responsible for DDIs at this level.

## 6. Excretion

Mass balance studies with [^14^C]-ATS have revealed the biliary route as the major route of elimination of ATS and its metabolites, with a minor contribution of the renal excretion (1–2%) to the overall elimination of the drug [3,22]. In fact, renal impairment has no significant effect on the PK parameters of ATS [42], which helps the management of complex dyslipidemia in hypercholesterolemic hemodialysis patients since no dose adjustment is required [49]. It has been demonstrated that ATS can be reabsorbed from the bile, thus suggesting biliary recycling as an important component in ATS metabolism and excretion and may contribute to the prolonged duration of ATS effect [43].

The involvement of P-gp in the absorption of ATS has been demonstrated in vivo due to the influence of polymorphisms in *ABCB1* genotypes [50]. However, the activity of P-gp affects the PK during the elimination phase more than in the absorption phase, as AUC and half-life (t_1/2_) show greater differences (*p* < 0.05), rather than C_max_ values, between genotypes [50]. These results suggest that P-gp affects the enterohepatic circulation of ATS.

## 7. Physiologically Based Pharmacokinetic Models of Atorvastatin

The PK characteristics of ATS have led to the development of PBPK models that can better explain the complexity of each of the LADME processes of this drug. The PBPK models of ATS published until now are described below.

### 7.1. Zhang, 2015

This is the first PBPK model of ATS that assess not only the parent drug but also the two main metabolites of the open acid form, ATS-L and 2OH-ATS [51]. This model is intended to evaluate DDIs between ATS and its metabolites at multiple scenarios (e.g., concomitant administration of enzyme inhibitors or inducers such as itraconazole, clarithromycin, cimetidine, rifampicin, and phenytoin). The PBPK model incorporates the Advanced Dissolution, Absorption and Metabolism (ADAM) model to characterize the absorption process and a full PBPK distribution model for predicting volume of distribution at steady state (V_ss_) and tissue-to-plasma partition coefficients (K_p,t_). The intestinal efflux process is implemented using in vitro determined maximum rate of transporter-mediated efflux (J_max_) and K_m_ of P-gp. The elimination of the open acid form occurs minimally through renal excretion (CL_R_ = 0.47 L/h), being the metabolic pathway the most important for ATS clearance. Enzymatic processes are mediated by CYP3A4 (*ortho*- and *para*-hydroxylation) and CYP2C8 (*para*-hydroxylation) processes and glucuronidation reactions mediated by UGT1A1 and UGT1A3. It must be noted that ATS hydroxylation by CYP3A4 incorporated *ortho*- and *para*-hydroxylation, and the resulting metabolite for the *ortho*-hydroxylation was its active metabolite (2OH-ATS). However, an important issue here is raised, as the in vitro assays of ATS metabolism reveal lactonization as the critical first step in ATS disposition and suggest that ATS-hydroxylated metabolites would be mostly formed after hydrolysis of the corresponding lactone products (2OH-ATS-L and 4OH-ATS-L) due to the higher affinity of the lactone form of ATS for the active site of CYP450 isoforms [39]. In this sense, the ATS *ortho*-hydroxylation may be overestimated (Inter System Extrapolation Factors (ISEF) of 7) to reproduce 2OH-ATS levels, and the resulting predicted CL of 51 L/h largely exceeds that observed after IV administration (37.5 L/h), with the corresponding lower predicted bioavailability (7% vs. 14%). Additionally, the 2OH-ATS product is not assessed by the model, but the corresponding formation pathway parameters are remarkably close to that of the *ortho*-hydroxylation route (ISEF of 7). This issue will produce higher amounts of 4OH-ATS than observed and probably causing an over-prediction of ATS systemic clearance. A permeability-limited liver model was used to assess hepatic OATP1B1-mediated active uptake and incorporated passive diffusion clearance in the hepatocytes membrane. Different ISEFs were applied to best reproduce the observed data, a well-known approach for in vitro/in vivo extrapolations (IVIVE) in enzymatic and transporter-mediated processes. CYP3A4-mediated *ortho*-hydroxylation and UGT1A3-mediated glucuronidation generate the primary metabolites 2OH-ATS and ATS-L, respectively, which are modelled through minimal PBPK distribution models using a Single Adjusting Compartment (SAC). As expected, the predicted V_ss_ of ATS-L was higher than the V_ss_ of ATS and 2OH-ATS. Despite the octanol:water partition coefficients are not different enough to justify this difference, the absence of the carboxylic acid functional group of the lactone form and the closed ring of its structure (neutral compound) may increase the permeability through cell membranes thus increasing its volume of distribution. As there were some physicochemical properties that were not available for these metabolites (e.g., B/P and f_u_), ATS corresponding parameter values were assumed. Elimination of both metabolites was parameterized through enzyme kinetics, determining CYP3A4-intrinsic clearance with the retrograde model after assigning 40% and 30% contribution of CYP3A4 to the overall clearance of 2OH-ATS and ATS-L, respectively. The metabolized fractions of both metabolites were assigned to reproduce observed clinical DDIs, so they can be considered when assessing their elimination. However, in the case of ATS-L a value of 1892 mL/min/mg protein for its intrinsic clearance by HLMs has been published [40] that could have been used and optimized if necessary, in a more mechanistic manner.

Model performance was evaluated using 13 independent clinical trials (data from literature) after oral doses of 20 and 40 mg of ATS (Table 1). The prediction ability of the PBPK model was validated using DDIs clinical data with enzyme inhibitors and inducers.

There are some aspects that need further consideration regarding the involvement of P-gp and hepatic transporters. The authors stated that ATS exhibits high solubility and high permeability, considering the contribution of P-gp to the total exposure to be marginal. However, the involvement of P-gp in ATS PK has been demonstrated in humans due to the polymorphisms in ABCB1 genotypes, thus suggesting that P-gp affects the enterohepatic recirculation of ATS [50]. Additionally, ATS is currently considered as a BCS class II (low solubility, high permeability), and many efforts have been made to increase its bioavailability enhancing its solubility [14,17,20,71,72]. For these reasons, solubility and P-gp activity become essential in ATS absorption and disposition. Furthermore, the contribution of hepatic transporters to ATS disposition cannot be minimal, as stated, because it has been demonstrated in vitro that transporter-mediated hepatic uptake clearly dominates overall ATS hepatic uptake with 90% ± 2% contribution [34]. This implication in ATS exposure has been demonstrated in vivo [35,37]. Despite this, the PBPK model of Zhang accurately described the time course of ATS, 2OH-ATS, and ATS-L after the oral administration of 40 mg of ATS in more than 10 independent clinical studies as well as DDIs with enzyme inhibitors and inducers, being the model more accurate to reproduce changes in AUC than in C_max_. Some of the limitations assumed by the authors are the lack of parameterization of the hydrolysis process of the lactone forms to the corresponding open acids and the optimization of the OATP1B1 kinetic parameters with only data from single-dose studies with rifampicin.

### 7.2. Duan et al., 2017

In this work [73], Duan et al. developed an ATS PBPK model to assess the role of OATP1B1 in ATS disposition evaluating *SLCO1B1* polymorphisms and the impact of DDIs on ATS exposure when co-administrated with known CYP and/or transporter inhibitors such as rifampicin, cyclosporine, gemfibrozil, itraconazole, and erythromycin. The authors used different data sets to develop and evaluate the model using data from the literature. The absorption process was modelled using the ADAM model, predicting human effective permeability (P_eff,man_) from Caco-2 cells. A full PBPK approach was considered as the distribution model, using the Rodgers and Rowland method [74,75] to predict K_p,t_ and V_ss_. Metabolism was only modelled via CYP3A4 oxidation, calculating CL_int,CYP3A4_ by means of two approaches: the first one used the retrograde methodology with the reported IV ATS clearance of 37.5 L/h and assuming 100% contribution of CYP3A4 to overall metabolic clearance, resulting in an intrinsic clearance of 8 mL/min/pmol recombinant CYP; the second one directly used intrinsic clearance from in vitro assays and accounted for *ortho*- and *para*-hydroxylation pathways. The CL_int,T_ for OATP1B1 obtained with both approaches were optimized starting from the reported in vivo CL_int,T_ of 910 mL/min/kg or 360 mL/min/million cells (based on SimCYP Simulator extrapolation algorithms), which was further decomposed in J_max_ and K_m_. Both approaches provided similar simulated ATS profiles and required the optimization of CL_int,OATP1B1_ with a scaling factor of 4 to best reproduce C_max_ and AUC of the training dataset. These results led to the consideration that hepatic uptake seems to be the rate-limiting step in ATS elimination, which constitutes an important conclusion of this work. No other CYP (CYP2C8 nor CYP3A5) was considered to contribute to ATS metabolism, despite the well-known metabolic profile previously described. In addition, no lactonization process was implemented in the model, which constitutes and important limitation (assumed by the authors) due to the relevance of the lactone forms of ATS and its metabolites, not only in the metabolic process [39] but also in terms of safety and toxicity [2,76]. The absence of these processes could explain the slight deficiency to accurately reproduce the terminal phase of the 20 and 40 mg oral dose of ATS. Regarding the distribution model, a V_ss_ of 0.226 L/kg was predicted by the model, which was notably lower compared to previously reported values of 381 L [3], 5.4 L/kg [24] and 8.7 L/kg [51]. Therefore, model parameter optimizations with observed PK parameters that are largely influenced by volume of distribution (e.g., C_max_) should be considered with caution. This PBPK model accounts, for the first time, for Breast Cancer Resistance Protein (BCRP) contribution to ATS disposition, and it is in line with the available data published by Keskitalo et al. [35] (subjects with the *ABCG2* c.421AA genotype showed a 72% and 46% increase in ATS AUC and C_max_, respectively, when compared to subjects with the c.421CC genotype). These results, as well as the unchanged t_1/2_ in both genotypes, suggested that the main role of BCRP is linked to the absorption phase. For this reason, intestinal BCRP activity was manually optimized to best fit C_max_ and T_max_ of the training dataset. BCRP canalicular efflux activity was also incorporated into the model, thus contributing to the biliary excretion of ATS, directly adopting the reported in vitro value of 1.4 mL/min/mg protein. A small contribution to overall elimination of ATS through the kidneys was also implemented.

Model performance was determined assessing the ratio of simulated (AUCR_sim_) and observed (AUCR_obs_) results of AUC with and without the perpetrator drug of the DDI (AUCR_sim_/AUCR_obs_) according to the two-fold range (0.5–2) due to the known inter-study variability.

The PBPK model developed by Duan et al. accurately described the time course of single oral doses of 20 mg and 40 mg of ATS in healthy volunteers. The PBPK model was able to capture the AUCRs in *SCLO1B1* polymorphism (c.521CC vs. c.521TT) and in the presence of CYP3A4 or OATP1B1 inhibitors reasonably well (ratios within 2-fold of the observed value).

### 7.3. Li et al., 2019

Li et al. refined the published ATS and ATS-L PBPK models by Zhang (2015) incorporating biliary excretion of ATS and OATP1B3-mediated hepatic active uptake [77]. The aim of the work was to assess in silico the potential of severe and life-threating myopathies such as rhabdomyolysis when ATS is concomitantly administered with CYP3A4 and/or OATP inhibitors. Absorption and distribution processes were modelled using ADAM and full PBPK models, respectively, incorporating a permeability-limited liver model to deal with hepatic transporters. Enterohepatic recirculation of ATS was allowed, but the fraction available to be reabsorbed was not reported. Metabolism of both ATS and ATS-L was enzymatically modelled using V_max_ and K_m_ values of CYP3A4 *ortho*- and *para*-hydroxylation. Another important feature of this model is that renal excretion was not implemented, and thus ATS elimination was restricted to the liver. Passive diffusion (CL_PD_) of ATS in the membrane of hepatocytes was assumed to be consistent with in vitro results, while CL_int,T_ was estimated through the Parameter Estimation module, and each of the transporters involved in hepatic active uptake were assigned to contribute almost equally to the total intrinsic clearance (53% and 47% contribution for OATP1B1 and OATP1B3, respectively). It is true that OATP1B1 and OATP1B3 are the main transporters in ATS hepatic active uptake [34], but the protein expression levels are quite different (23.2 vs. 3.2 fmol/µg membrane protein for OATP1B1 and OATP1B3, respectively), as well as V_max_ (OATP1B1 V_max_ is three-fold higher than OATP1B3 V_max_). Thus, the assigned role of each of these transporters should be considered with caution. The PBPK model did not incorporate other transporters responsible of ATS hepatic active uptake (e.g., NTCP and OATP2B7) that had also demonstrated in vitro activity [34]. On the other hand, the ATS-L model needed the incorporation of empirical scaling factors to the metabolic pathways and an additional liver microsomal clearance to best fit the observed data. Sensitivity analysis on this additional clearance mechanism is lacking.

Model verification was performed by comparing simulated PK parameters AUC and C_max_ with observed data at different dose levels (10, 20, and 40 mg). Despite all simulations were within the desired two-fold error range, AUC predictions were, in general, overpredicted, while C_max_ was less variable and closer to the observed values. This situation could be explained due to the absence of implementation of other metabolic pathways in the elimination of both ATS and ATS-L, such as CYP3A5 and CYP2C8-mediated hydroxylation. Model validation was performed using DDI studies with ATS (and ATS-L) as victim drug and inhibitors (perpetrators) of CYP3A4 (itraconazole and clarithromycin), OATP1B1 and OATP1B3 (rifampicin) or both (cyclosporine). Results revealed an important feature of ATS PK: ATS lactonization must not be an immediate process, and the intermediate of the acid-to-lactone conversion, i.e., acyl-β-D-glucuronide, should be present in the bloodstream sufficient enough to be victim of OATP-mediated transporter inhibition to increase ATS-L exposure more than three times when co-administered with rifampicin and cyclosporine. Thus, UGT-mediated glucuronidation is the main route of ATS lactonization, and this metabolic pathway should be considered when developing ATS PBPK models. Finally, model application to predict ATS and ATS-L exposure in muscle tissues revealed that ATS-L levels are 18-fold higher than in plasma and 10- or 14-fold higher than ATS exposure in muscle tissues after single or multiple doses of 40 mg. These results are consistent with the high V_ss_ of ATS-L predicted by the model (141.3 L/kg).

However, the model has some limitations that are recognised by the authors and are summarized as follows: (i) the lack of other recognised transporters implicated in ATS disposition such as MDR1 (P-gp) and BCRP; (ii) the absence of pre-systemic non-enzymatic ATS lactonization at initial segments of the GI tract; (iii) the impossibility to describe the kinetics of the glucuronide intermediate in the acid-to-lactone conversion.

### 7.4. Morse et al., 2019

This is the first PBPK model that considers pre-systemic degradation of ATS [78]. The model developed by Morse et al. states that ATS-L is mostly formed non-enzymatically in the stomach due to the low pH of this region of the GI tract. Taking this premise in mind, the authors developed a PBPK model to assess the impact of increasing gastric emptying time after glucagon-like peptide-1 receptor agonists (GLP1RAs) administration. Due to the limitation of considering two active drugs as substrates in Simcyp v17, two PBPK models were simultaneously developed: one considering ATS as substrate and a second model considering ATS-L as substrate. This is made by dividing the oral dose of ATS as a function of the fraction absorbed (f_a_) of ATS, since complete absorption of ATS has been suggested [3]. The pre-systemic degradation is performed optimizing a stomach degradation rate constant to reproduce the dose-dependent change in C_max_, which is observed in clinical studies. The fraction absorbed of ATS derived from the best scenario is considered to determine ATS-L f_a_. This strategy allowed to manage a fraction of the dose administered directly as ATS-L and generating the corresponding substrate model file, thus assessing its ADME properties more mechanistically. To better characterize ATS pre-systemic degradation, pH-dependent solubility was added to the model, taking a dissolution profile directly from the literature [44]. However, it must be noted that this pH-dependent dissolution profile has been characterized for the sodium salt of ATS, and at present ATS is administered as the tri-hydrated calcium salt of the carboxylic acid, which could lead to differences in the dissolution profile when compared to clinical data. The ADAM model is used for assessing the absorption of both substrates predicting P_eff,man_ from Caco-2 cells and MDCK for ATS and ATS-L, respectively.

The model structure considers 2OH-ATS as a direct metabolite of ATS and a secondary metabolite of ATS-L due to plasmatic hydrolysis of 2OH-ATS-L after CYP-mediated hydroxylation. ATS is also considered in the ATS-L model as a direct metabolite after plasmatic esterase-mediated hydrolysis. ATS and ATS-L metabolism are parameterized by enzyme kinetics mainly through CYP3A4, although an additional non-CYP microsomal intrinsic clearance was optimized to best reproduce the in vivo interaction with itraconazole. Additionally, UGT1A3-mediated metabolism is also implemented for ATS, but it does not generate the corresponding lactone product. No other CYPs nor UGTs are considered. Plasmatic hydrolysis of lactone forms (ATS-L and 2OH-ATS-L) to the corresponding acid products (ATS and 2OH-ATS) are included in the model in terms of half-life (minutes) for ATS-L and through an esterase intrinsic clearance (µL/min/mg) for 2OH-ATS-L to best fit the observed data after an oral dose of 40 mg, and this was verified with DDIs studies with itraconazole and dulaglutide. Additionally, sensitivity analyses on optimized parameters such as esterase activity and non-CYP-mediated hepatic metabolism were performed. An important feature of this work is that some PBPK model parameters were determined in vitro, such as CL_int,OATP1B3_ (µL/min/10^6^ cells) for ATS and 2OH-ATS (31.5 and 25, respectively), P_app_ (10^−6^ cm/s) for ATS-L in MDCK cells (33) and CL_PD_ (no units provided) for ATS and 2OH-ATS (13 and 5, respectively), thus avoiding some assumptions and optimizations, and increasing model identifiability [79].

Different distribution models are selected for each moiety, and a K_p_ scalar is applied to best reproduce C_max_ (ATS) or t_1/2_ (ATS-L and 2OH-ATS-L) at 40 mg dose level. ATS model predicted V_ss_ is, as in the PBPK model developed by Duan et al., quite lower (0.69 L/kg) than those previously published [51,77], so caution must be paid to the values of the model parameters optimized, with observed PK parameters largely influenced by the volume of distribution. ATS-L exhibits a higher V_ss_ (18.182 L/kg), which is in line with its higher lipophilicity and neutral acid-base properties. Permeability-limited liver model is only used for ATS and 2OH-ATS, which incorporates a hepatic OATP1B3-mediated active uptake process optimized through a scaling factor. Biliary excretion, as well as sinusoidal efflux, of 2OH-ATS is added to the model with the corresponding optimized intrinsic clearance values. Model performance was finally verified comparing simulated PK parameters AUC, C_max_, and T_max_, with those obtained in clinical DDI studies with itraconazole and the GLP1RA dulaglutide.

The model developed by Morse et al. fills an important gap of the above PBPK models of ATS as it accounts for a non-enzymatic lactonization process due to the low pH of the stomach that takes place pre-systemically and is responsible for the rapid appearance of ATS-L in plasma (T_max_ range 2–3 h). In this line, a potential novel DDI with proton pump inhibitors (PPIs) has been robustly identified and associated with increased plasma concentrations of ATS, 2OH-ATS, ATS-L, and 2OH-ATS-L [80]. Despite some PPIs such as omeprazole and lansoprazole are known CYP substrates and enzyme inhibitors, the increase in the exposure was explained through an increase in ATS bioavailability secondary to an increase in ATS solubility and a decrease in the pre-systemic lactonization due to the PPI-induced rise in gastric pH.

## 8. Discussion

The development of PBPK models, commonly known as “bottom-up approach”, largely rely on previously gathered in vitro and/or in vivo information to build up the mechanisms able to reproduce the experimental evidence from clinical trials. Minimal parameter estimation/optimization is, therefore, required to characterize the time-course of the analyte(s). In this sense, adequate external experimental evidence is needed in order to properly assess the mechanisms implemented in order to use the PBPK as a predictive tool for dose optimization in patients and/or special sub-groups of populations. The published PBPK models of ATS were developed using an ATS dose range between 10 and 40 mg, but no information was incorporated into the models of the highest dose strength (80 mg). Additionally, clinical trials incorporated mainly healthy volunteers (*n* = 434) with no information regarding patients with hypercholesterolemia. Moreover, most of the clinical trials were conducted after single-dose regimens (*n* = 17), whereas limited evidence was gathered after multiple-dose regimens (*n* = 3). The multiple PK pathways affecting ATS could be partially influenced due to disease status and chronic administration of ATS. This limitation could impact the simulation-based dose selection in patients.

The low solubility of ATS is a major drawback affecting its absorption and, consequently, its bioavailability. The lack of adequate reported information regarding the solubility profile of ATS-Ca represents a limitation in the development of mechanistic models of ATS dissolution throughout the GI tract. Available information on the solubility profile of ATS-Na [44] could be used as a provisional input during model development, but additional efforts should be performed to properly provide experimental evidence about ATS-Ca in this regard. Due to the multiple processes affecting ATS-Ca within the GI lumen (lactonization, hydrolysis, drug dissolution), a detailed characterization of its solubility would enhance the prediction of its bioavailability and, therefore, the evaluation of new oral formulations of ATS incorporating mechanisms improving the solubility of ATS-Ca.

A relevant aspect that Li et al. incorporated into the PBPK model is the assumption that ATS lactonization might be a non-immediate process within the bloodstream, which was observed when OATP-mediated transporter inhibitors were co-administered with ATS. Acyl-β-D-glucuronide is an intermediate compound during the lactonization process, and it is a substrate of OATP transporters for hepatic uptake, which is inhibited in the presence of compounds with higher affinity to OATP transporters (i.e., rifampicin and cyclosporine). The kinetic equilibrium in plasma is, therefore, displaced to the more lipophilic form (ATS-L), affecting the distribution into low-perfused tissues. On the other hand, the PBPK model developed by Morse et al. incorporates the alternative lactonization pathway that occurs at low pH values (stomach). This process explains the rapid appearance of ATS-L in plasma due to pre-systemic lactonization. The higher permeability of ATS-L because of its higher lipophilicity compared to ATS enables a shorter T_max_ in plasma. In this sense, the incorporation of the lactonization process (UGT-mediated) is clearly required to properly characterize the disposition of ATS and ATS-L in plasma and other tissues.

Simcyp simulator is one of the most used PBPK software currently available, with scientific [81,82,83] and regulatory [79,84,85,86] agreement for the establishment of quantitative PBPK frameworks, allowing dose selection in special sub-groups of populations, DDI and transporter evaluation, and biopharmaceutical specification characterization. Nevertheless, the development of a PBPK model able to simultaneously predict the PK profile of the six analytes (ATS, 2OH-ATS, 4OH-ATS, ATS-L, 2OH-ATS-L, and 4OH-ATS-L) with the current version of Simcyp (v20) represents a major challenge, since it only allows to consider simultaneously one parent drug (substrate), two primary metabolites, and a secondary metabolite. Considering the lactonization/hydrolysis equilibrium and the parallel formation of metabolites from ATS and ATS-L, the prediction ability of the PBPK model is clearly affected when more than four analytes are considered.

To the best of our knowledge, these are the four PBPK models of ATS published (Table 2). All of them consider ATS as a lipophilic monoprotic acid of 559 g/mol with a pK_a_ of about 4.4. The ADAM model is used to characterize the absorption of the drug and predict the human permeability from Caco-2 cell experiments. Full PBPK distribution models for ATS are used in all of them, being the Rodgers and Rowland method (#2) the most used to predict V_ss_ and K_p,t_. This selection agrees with the physicochemical properties of ATS because of its degree of ionization at physiological pH (pKa ≈ 4.4), as this method can deal with the different fractions of the drug (ionized or non-ionized). Metabolism is modelled through enzyme kinetics in all cases and mainly by means of CYP3A4. However, some models use another CYP isoform (CYP2C8 in Zhang, 2015) or an unspecific metabolism through HLM (Morse et al., 2019). Lactonization is modelled enzymatically through UGT1A1 and UGT1A3 in the models of Zhang and Li et al., while it is considered to occur non-enzymatically in the stomach in Morse et al. As both processes have been demonstrated to contribute to ATS-L formation, any PBPK model of ATS should consider them to best reproduce the PK of ATS. Regarding transport processes, it must be noted that all the models use the Permeability Limited Liver Model to account for active transport added to the passive diffusion through the cell membranes of the hepatocytes. OATP1A1 is incorporated in all the models, while OATP1B3 is only considered in the models of Li et al. and Morse et al. Efflux processes are implemented in the gut wall through P-gp and BCRP only in the model of Zhang, and a canalicular efflux process mediated by BCRP is assessed only in the model of Duan et al. As stated previously, the roles of P-gp [50] and BCRP [35] in the PK of ATS have been demonstrated in vivo, thus, the PBPK models of ATS should incorporate them to best characterize its absorption and enterohepatic recirculation. Renal excretion of ATS contributes to the overall elimination of the drug to a lesser extent; thus, its presence in PBPK models is not mandatory. For this reason, only two of the four models implemented it. However, as enterohepatic recirculation has been suggested (and demonstrated in pre-clinical species [43]), bile excretion is a route that could be considered to best characterize this process as it occurs with the models of Duan et al. and Li et al.

According to Table 2, there is no consensus between models regarding physicochemical parameters such as logP_o:w_ (ranging from 4.07 to 5.7) and f_u_ (values from 2.2 to 5.1%). As a result, different V_ss_ values arise, ranging from 0.226 to 8.7 L/kg, despite using the same method for its prediction. Therefore, both parameters (logP_o:w_ and f_u_) influence the V_ss_ obtained. However, in the case of V_ss_ predicted by the model of Li et al. and Morse et al., the Lipid Binding Scalar overweights the logP_o:w_ and enhances the distribution of ATS. B/P is homogeneous in all models with the exception of that of Morse et al., in which the default value was 0.55 and no distribution into red blood cells was assumed. P_eff,man_ is another parameter highly variable between models depending on the value of the P_app_ introduced in the platform and the conditions of the in vitro experiment.

P-gp activity is only assessed in the model of Zhang, suggesting little confidence on the relevance of this transporter in ATS PK. Several studies with in vitro models have demonstrated that ATS is an inhibitor of P-gp and may be a substrate of this transporter [88]. Additionally, concomitant administration of 80 mg of ATS with digoxin increased digoxin AUC_0–24_ and C_max_ by 15% and 20%, respectively [89]. As neither digoxin T_max_ nor renal clearance were affected, it has been suggested that the mechanism of this DDI is the inhibition of the P-gp-mediated intestinal efflux of digoxin by ATS. Thus, modelling of P-gp-mediated transport processes in ATS PBPK is important to better characterize potential DDIs at this level not only as a perpetrator, but also as a victim drug, to avoid high exposures that could lead to the development of adverse events such as myopathies. The model of Duan et al. confirms that ATS hepatic uptake by members of the OATP family is the rate-limiting step in ATS elimination, as previously described. However, lactonization is not implemented in this model, which constitutes an important limitation to better characterize ATS metabolism.

The relative contribution of the hepatic uptake transporters OATP1B1 and OATP1B3 is assigned almost equally (53% and 47%, respectively) in the model of Li et al. after estimating total intrinsic uptake clearance instead of using enzymatic or intrinsic clearance values determined in vitro as Zhang, Duan et al., and Morse et al. did. Because protein expression levels and V_max_ of these transporters are quite different, this statement should be managed with caution when developing a PBPK model of ATS.

## 9. Conclusions

The development of solid, physiologically based pharmacokinetic models clearly enhances the decision-making process, helping to understand and infer how PK processes may affect the optimal posology in the target population. Several aspects have been highlighted as critical elements in the complex pharmacokinetics of atorvastatin that could compromise its efficacy/safety in patients with hypercholesterolemia: (i) the integration of the lactonization process, which occurs within the GI lumen and plasma, and represents a major kinetic process that affects the formation of additional active moieties (2OH-ATS and 4OH-ATS); (ii) the contribution of P-gp has been undermined in most of the PBPK models developed so far, limiting the evaluation of DDI effects; (iii) the varying effect of ATS-Ca solubility within the GI tract; (iv) the inclusion of additional experimental evidence in patients and multiple regimen schedules; and (v) the simultaneous management of multiple analytes within the PBPK platforms in order to optimize the benefit/risk balance of ATS.

## Figures and Tables

**Table 1:** Study design and population characteristics of the physiologically based pharmacokinetic models of atorvastatin.

	Zhang, 2015	Duan et al., 2017	Li et al., 2019	Morse et al., 2019
Number of independent clinical studies	13 ^a^	7 ^b^	6 ^c^	5 ^d^
Dosing regimen (Number of trials)	SD (12) MD (2)	SD (6) MD (2)	SD (5) MD (1)	SD (5)
Number of subjects (total)	386	166	180	145
Clinical status (number of subjects)	HV (386)	HV (145) RTP (21)	HV (162) RTP (18)	HV (145)
Dose level (mg) (number of subjects)	20 (83) 40 (303)	10 (33) 20 (60) 40 (73)	10 (36) 20 (55) 40 (89)	10 (12) 40 (133)

^a^: [52,53,54,55,56,57,58,59,60,61,62,63,64]; ^b^: [52,54,60,61,65,66,67]; ^c^: [35,59,60,64,68,69]; ^d^: [59,60,67,69,70]; SD: single dose schedule; MD: multiple dose schedule; HV: healthy volunteers; RTP: renal transplant patients.

**Table 2:** Parameters of each physiologically based pharmacokinetic model of atorvastatin.

Model Parameter	Zhang, 2015	Duan et al., 2017	Li et al., 2019	Morse et al., 2019
Physicochemical Properties				
Molecular weight (g/mol)	558.66	558.64	558.64	559.00
logP_o:w_	5.7	4.07	4.434	5.39
Compound type	Monoprotic Acid	Monoprotic Acid	Monoprotic Acid	Monoprotic Acid
pK_a_	4.46	4.46	4.46	4.33
B/P	0.61	0.61	0.61	0.55
f_u_	0.051	0.024	0.050	0.022
Absorption				
Model	ADAM	ADAM	ADAM	ADAM
P_eff,man_ (10^−4^ cm/s)	2.05	NR	1.05	4.49
In vitro assay	Caco-2	Caco-2	Caco-2	Caco-2
pH_apical_:pH_basolateral_	7.4:7.4	7.4:7.4	7.4:7.4	6.5:7.4
P_app_ (10^−6^ cm/s)	8.6	7.9	4.9	28.4
Refference compound	Propranolol	NR	NR	NR
P_app refference_ (10^−6^ cm/s)	20	NR	NR	NR
Distribution				
Model	Full PBPK	Full PBPK	Full PBPK	Full PBPK
Method	1 and 2	2	2	2
V_ss_ (L/kg)	8.7	0.226	2.67	0.690
Kp scalar (model)	2(1) and 4.6(2)	NR	NR	2
Lipid Binding Scalar	NR	NR	4.15	NR
Metabolism				
Model	Enzyme kinetics	Enzyme kinetics	Enzyme kinetics	Enzyme kinetics
**CYP3A4**				
Metabolite	2OH-ATS			2OH-ATS
K_m_ (βM)	29.7		34.8	34.8
V_max_ (pmol/min/pmol isoform)	29.3		1048	1048
f_u,mic_	1		NR	NR
Scaling Factor	7 (ISEF)		NR	NR
K_m_ (µM)	25.6		33	33
V_max_ (pmol/min/pmol isoform)	29.8		1353	1353
f_umic_	1		NR	NR
CL_int_ (µL/min/pmol isoform)		8	NR	NR
Scaling Factor	7 (ISEF)			
**CYP2C8**				
K_m_ (µM)	35.9			
V_max_ (pmol/min/pmol isoform)	0.29			
f_u,mic_	1			
Scaling Factor	4 (ISEF)			
**UGT1A1**				
Metabolite	ATS-L		ATS-L	
K_m_ (µM)	11		2	
V_max_ (pmol/min/pmol isoform)	72		2	
f_u,mic_	1		NR	
Scaling Factor	2 (ISEF)		NR	
**UGT1A3**				
Metabolite	ATS-L		ATS-L	
K_m_ (µM)	11		4	
V_max_ (pmol/min/pmol isoform)	72		38	
f_u,mic_	1		NR	
Scaling Factor	2 (ISEF)		NR	
CL_int_ (µL/min/mg protein)				6.2
**Other HLM**				
CL_int_ (µL/min/mg protein)				65
*Transport*				
*Intestine*				
**P-gp**	Efflux (gut wall)			
K_m_ (µM)	115			
J_max_ (pmol/cm^2^/min)	141			
Scaling Factor	1 (RAF/REF)			
**BCRP**	Efflux (gut wall)			
CL_int,T_ (µL/min)	6			
*Liver*				
CL_PD_ (mL/min/10^6^ cells)	0.023	0.017	0.023	0.013
f_u,IW_	0.324			
f_u,EW_	0.038			
**OATP1B1**	Uptake (sinusoidal)			
CL_int_ (µL/min/10^6^ cells)			1000	31.5
CL_int,T_ (µL/min)	55			
K_m_ (µM)		0.77		
J_max_ (pmol/min/10^6^ cells)		277.2		
Scaling Factor	10 (RAF/REF)	4		30
**OATP1B3**	Uptake (sinusoidal)			
CL_int_ (µL/min/10^6^ cells)			900	31.5
Scaling Factor				30
**BCRP**	Efflux (canalicular)			
CL_int,T_ (µL/min/10^6^ cells)		1.4		
*Excretion*				
CL_R_ (L/h)	0.47	0.375		
CL_int,bile_ (µL/min/10^6^ cells)			10	

Green: data from literature; Blue: in situ determined value; Yellow: predicted value; Orange: optimized value according to observations; Red: assumed value; B/P: blood-to-plasma ratio; f_u_: fraction unbound in plasma; ADAM: Advanced Dissolution, Absorption and Metabolism model; P_eff,man_: human effective permeability; NR: not reported; P_app_: apparent permeability; Method 1: Poulin and Theil method [87]; Method 2: Rodgers and Rowland method [74,75]; V_ss_: volume of distribution at steady state; K_m_: Michaelis–Menten constant; V_max_: maximum rate of the enzymatic process; f_u,mic_: fraction unbound in the microsomal incubation; CL_int_: intrinsic clearance; HLM: human liver microsomes; P-gp: P-glycoprotein; J_max_: maximum transport rate of the transporter; BCRP: breast cancer resistance protein; CL_int,T_: total intrinsic clearance of the transporters; CL_PD_: passive diffusion clearance through cell membranes; f_u,IW_: fraction unbound in the intracellular water; f_u,EW_: fraction unbound in the extracellular water; CL_R_: renal clearance; CL_int,bile_: biliary intrinsic clearance.

## Data Availability

Not applicable.
